# Barriers to the digital transformation of infrastructure sectors

**DOI:** 10.1007/s11077-021-09438-y

**Published:** 2021-11-03

**Authors:** Liliane Manny, Mert Duygan, Manuel Fischer, Jörg Rieckermann

**Affiliations:** 1 Institute of Civil, Environmental and Geomatic Engineering, ETH Zürich, Zurich, Switzerland; 2 Eawag, Swiss Federal Institute of Aquatic Science and Technology, Dübendorf, Switzerland; 3 Institute of Political Science, University of Bern, Bern, Switzerland; 4 Faculty of Business and Economics, University of Basel, Basel, Switzerland; 5 Laboratory on Human-Environment Relations in Urban Systems, EPFL ENAC IIE HERUS, Lausanne, Switzerland

**Keywords:** Digital transformation, Data utilization, Infrastructure, Wastewater, Switzerland, QCA

## Abstract

Digital technologies can be important to policy-makers and public servants, as these technologies can increase infrastructure performance and reduce environmental impacts. For example, utilizing data from sensors in sewer systems can improve their management, which in turn may result in better surface water quality. Whether such *big data* from sensors is utilized is, however, not only a technical issue, but also depends on different types of social and institutional conditions. Our article identifies individual, organizational, and institutional barriers at the level of sub-states that hinder the evaluation of data from sewer systems. We employ fuzzy-set Qualitative Comparative Analysis (fsQCA) to compare 23 Swiss sub-states and find that two barriers at different levels can each hinder data evaluation on their own. More specifically, either a lack of vision at the individual level or a lack of resources at the organizational level hinder the evaluation of data. Findings suggest that taking into account different levels is crucial for understanding digital transformation in public organizations.

## Introduction

Digitalization refers to a multiplicity of on-going processes in many different sectors, whereby the implementation of digital technologies and the utilization of obtained data constitutes a unifying element. Digital transformation goes beyond digitalization, as it additionally includes organizational and institutional changes, e.g., organizational culture, regulation, or service delivery, and affects the public sector, among other sectors (Giest, 2017; Mergel et al., 2019). Digital transformation is also gaining ground in the domain of public infrastructure (Apráez & Lavrijssen, 2019). An increasing number of municipal and regional authorities have started experimenting with applications that rely on information and communication technologies (Guenduez et al., 2018).

The increased application of sensors and data transmission technologies enables more sophisticated instrumentation and highly accurate measurements, generates big data that is large in volume, features incoming real-time data streams, and requires the employment of advanced analytics or algorithms (Klievink et al., 2016). Despite concerns over data security and over-reliance on data for the resolution of complex problems (Giest & Samuels, 2020), big data is also acclaimed for providing an important potential for higher performance (Rogge et al., 2017; Vydra & Klievink, 2019). For instance, the public water utility Denver Water has started to use big data to proactively identify trends that could point out to potential system failures before they arise as problems (Heaton, 2013). Big data is also used by the public transportation authority of Singapore to reduce crowding in congested areas (Maciejewski, 2016). Studies have further analyzed the rollout of smart meters for consumers in the energy sector (de Reuver et al., 2016).

Despite potential benefits, the utilization of big data is still limited and only recently gaining attention, especially in the public sector (Klievink et al., 2016). The literature focusing on social conditions influencing digital transformation has mostly studied just the adoption of new technologies, whereas conditions favoring or hindering the effective utilization of data have been overlooked (Maciejewski, 2016; Sun et al., 2016; Surbakti et al., 2019). Digitalization per se does not often provide immediate benefits, especially if the resulting big data is not utilized, and leads to data wasting (Mergel et al., 2016). As the volume of data produced by new technology is increasing exponentially, the need for data analytics, processing, and interpretation of data is also growing (Ingildsen & Olsson, 2016). However, the issue at hand goes beyond mere technical competence, but also involves organizational capabilities and readiness as the utilization of big data may require changes in roles (e.g., chief data officer as a new executive), routines, and decision-making within an organization (Klievink et al., 2016; Sun et al., 2016). For example, data from digital technologies may be included in performance indicators, therefore serving the purposes of reporting and the assessment of regulative targets (Lewis, 2015; Pollitt, 2013). The formulation of multiple indicators that reflect interests of different actors, as well as increased transparency (e.g., through public participation in the reporting process), could help reduce the performance paradox (i.e., poor reporting) in the public sector (Bolognesi & Pflieger, 2019; van Thiel & Leeuw, 2002), and ultimately improve infrastructure management and reduce environmental impacts.

In order to understand how public authorities utilize newly available data of infrastructure systems and what challenges impede its effective use (Giest & Raaphorst, 2018), we analyze and compare Swiss sub-states (Rieckermann et al., 2017). Our empirical focus lies on an oft-neglected and largely invisible infrastructure: sewer systems. Even though they involve high public investment costs, knowledge about the performance of these systems is only scarcely available as obtained data^1^ is rarely utilized (Rieckermann et al., 2017). Understanding barriers to the utilization of data in Switzerland’s urban water management practice is relevant for designing feasible policies for the handling of big data by public authorities, as well as on performance management of infrastructure systems. The digital transformation of sewer systems holds the potential to improve daily operational activities, the infrastructure planning process, as well as the protection of surface waters (Sarni et al., 2019).

Based on a combination of deductive and inductive approaches, the study identifies conditions that might act as barriers to the evaluation of data in Swiss sub-states. More specifically, we first discuss different strands of literature and deductively identify a general model with individual, organizational, and institutional levels, before relying on exploratory and semi-structured expert interviews to inductively specify four conditions applicable to our case. The four conditions and the outcome are operationalized based on in-depth interviews and document analysis, and—given that we expect conditions from the individual, organizational, and institutional levels to interact—cases are compared through Qualitative Comparative Analysis (QCA).

With this analysis, we make three contributions to the literature. First, we address a less researched but crucially important aspect of digital transformation, that is, the utilization of data (Maciejewski, 2016; Sun et al., 2016; Surbakti et al., 2019). By discerning the conditions impeding data utilization, our study sheds light onto the social and institutional barriers to digital transformation, which can be considered a radical innovation (Hage, 1980). Second, we bring together perspectives from policy and innovation studies, information technology, and environmental engineering in an interdisciplinary exercise. This results in a model of potential barriers to digital transformation including individual, organizational, and institutional levels, and potential interactions across them (Klievink et al., 2016; Sun et al., 2016; Surbakti et al., 2019). With respect to technological change in public organizations, these have not been jointly analyzed in the existing literature to date. Third, while many conceptual articles with illustrative examples (Giest, 2017; Lavertu, 2016) or single case studies (Barns et al., 2017; Giest & Raaphorst, 2018) deal with big data and politics, barriers to digital transformation have rarely been studied in a medium-N comparative setting with potentially important contextual variations across cases (Chatwin et al., 2019). Our empirical focus on urban water management provides an analysis of social and institutional barriers to the utilization of data by Swiss sub-state authorities. Overcoming such barriers could improve performance management of sewer systems and may result in higher efficiency, with improved economic and environmental outcomes.

The remainder of this article is structured as follows. In the next section, we compile insights from the literature with a focus on digital transformation and barriers at different levels. After introducing our specific research context in “Empirical setting: utilizing data from sensors in Swiss sewer systems” section, we combine deductive logic with inductive reasoning, drawing on our case knowledge and findings from expert interviews. We identify potential barriers to the evaluation of data from sewer systems which we describe in “Methods” section, along with the analytical method. We then present the results of our analysis followed by a discussion. The article ends with conclusions that discuss the transferability of results, limitations of the analysis, and future research questions.

## Politics, big data, and barriers to digital transformation

### Potential benefits and critical voices

Digital transformation can affect the entire policy cycle (Höchtl et al., 2016), from the input side of policy processes, through innovations in democratic participation (Janssen & Helbig, 2018), to its end, through the implementation of policies through public e-services or data-based evaluation of policies (Lavertu, 2016). The digital transformation has often been said to pave the way for more evidence-based policymaking in public administrations (Giest, 2017; Höchtl et al., 2016), or increased public infrastructure performance. For example, increased use of sensors and digital technologies enables real-time monitoring of traffic flow, public transportation, and air and water quality (Munné, 2016). Furthermore, data sharing can increase transparency and facilitate the participation of stakeholders or the broader public, as this information can be used to provide feedback or suggestions (Matheus et al., 2020). Finally, digital transformation can influence principal-agent relations (e.g., Wood and Waterman (1991); Maggetti and Papadopoulos (2018)) by affecting information (a)symmetries between, e.g., utilities and authorities. For all these reasons, concepts such as “open government” (Chatwin et al., 2019) or “digital-era governance” are argued to have replaced paradigms such as new public management (Dunleavy et al., 2005).

There is, however, a broad diversity of critical voices. As far back as 1972, Millar (1972) criticizes a potential bias toward mechanistic and abstract solutions for human problems, and a failure to properly take into account limited human and organizational abilities. Lavertu (2016) discusses the problems of potential misperceptions of organizational performance due to publicly available data to actors outside of public agencies. Likewise, complex, low quality, or inaccurate data can risk misinterpretation while data privacy issues can lead to mistrust (Matheus et al., 2020). Most recently, discussions have involved a potential bias against digitally illiterate or minority groups (Giest & Samuels, 2020).

### A general model of barriers to digital transformation

The disconnect between data production and data utilization (Giest & Ng, 2018) is not only reflected in these critical voices, but is further exacerbated by the fact the digital transformation also requires human and organizational capacities for data analytics, processing, and interpretation of data (Ingildsen & Olsson, 2016). While technical advancements in digital technologies promise a multiplicity of benefits, their implementation into existing organizational structures that would allow the actual reaping of the benefits is often challenging (Shearmur & Poirier, 2016; Wang & Feeney, 2014). Long-established structures and procedures of policymaking and implementation tend to be “sticky,” public administration actors generally rely on traditions and established “ways of doing things” (DiMaggio & Powell, 1983), and the stable mind-set of any organization will support only a limited range of innovations (Walker, 2006). Most innovations are in a continuum ranging from incremental to radical innovations rather than fully representing one (Hage, 1980). Digital transformation driven by the utilization of big data can be considered as rather a radical innovation.

Previous findings in innovation studies, public administration, management and information technology show that similarly to the adoption processes of technological innovations, the utilization of big data in organizations is contingent upon several conditions that can be attributed to three levels: individual, organizational, and institutional levels (Klievink et al., 2016; Sun et al., 2016; Surbakti et al., 2019). Similarly, the technology–organization–environment (TOE) framework claims the adoption of an innovation to be dependent on technological, organizational and environmental contexts (i.e., institutional and geographical structures) that the organization in question is part of (Tornatzky et al., 1990). Finally, also actor-centered institutionalism in political science or public administration studies (Scharpf, 2018) emphasizes that actors’ (individuals or organizations) rational behavior is influenced by the institutional context and cultural norms. We thus jointly analyze conditions at all three levels of individuals, organizations, and institutions, and expect that conditions on the three levels potentially interact and produce complex configurations of conditions. For example, a specific individual-level condition might only matter in one given institutional context.

### Individual-level conditions

Individual characteristics such as attitude, belief, and vision of managers can be highly important for organizational innovation (Rogers, 2003), including the use of big data (Corbett & Webster, 2015). Managers’ cognitive orientation and vision toward the perceived value of data-driven management is also referred to as “technology acceptance” (Venkatesh & Davis, 2000). Klievink et al. (2016) refer to this attribute as internal attitude of individuals, defined as “capability to develop internal commitment and vision for new processes and systems, especially openness toward data-driven decision-making” (p. 275). For example, a high level of employee engagement in terms of personal commitment and the presence of championing figures who actively promote big data is important for its use in organizations (Surbakti et al., 2019). Taking into account the perceptions of public managers helps assess why uses of big data remain very limited in the public sector (Guenduez et al., 2018; Mergel et al., 2018).

### Organizational conditions

The literature emphasizes two organizational conditions important for the innovativeness of an organization (Clausen et al., 2019). First, a lack of resource capacity to deal with data by relevant actors can represent a major bottleneck for digital transformation (Giest, 2017). Human resources are found as one of the most important conditions affecting organizations’ adoption of data utilization practices (Sun et al., 2016). More specifically, in case of big data, the adequacy of IT-literate personnel and availability of data science expertise are critical for organizations to develop a data use strategy (Klievink et al., 2016) and might be relevant to avoid disclosure biases and a performance paradox (e.g., Bolognesi and Pflieger 2019; van Thiel and Leeuw 2002; Lewis 2015). Factors comprising human capital, such as the number of employees or presence of specialized personnel, are especially important for radical innovations which involve fundamental changes that embed a high degree of new knowledge (Dewar & Dutton, 1986).

In addition to human resources, a data oriented innovation culture is essential for successful initiatives of big data utilization (Marshall et al., 2015; Surbakti et al., 2019). For instance, municipalities advanced in e-government initiatives are more likely to be the ones with higher in-house ICT (information and communications technology) activities and intranet infrastructure (Arduini et al., 2010). An established digitalization culture across the entire organization can indicate not only the readiness but also the experience required for incorporating data into internal planning and decision-making processes. Civil servants benefit from organizational support for their potential interest in digital transformation through, e.g., training or sharing of data among government departments (Giest, 2017). As many organizations still struggle to transition from paper-based management (Austin, 2018), the lack of an established digitalization culture is likely to impede the utilization of data.

### Institutional conditions

Organizations are embedded in several types of institutional contexts that influence them through expectations and cognitive frames and their social embeddedness and networks (DiMaggio & Powell, 1983; Williamson, 2000). Such institutional contexts and networks can also influence transaction costs (Andrews-Speed, 2016; Williamson, 2000), an important mechanism especially when dealing with data processing and interpretation. An increased diversity in size and number of partners with whom data is exchanged may correspond to an increased variety of data sources and formats, as well as different demands regarding organizational and coordinative efforts when it comes to receiving and treating data. This diversity may lead to a larger burden on authorities as one end user of data since merging data from diverse sources can require significant effort and time (Austin, 2018), and could again lead to disclosure biases and performance paradox (e.g., Bolognesi and Pflieger (2019); van Thiel and Leeuw (2002); Lewis (2015)). This institutional barrier has also been described as stemming from existing administrative and institutional structures define the way data is collected, analyzed, and used, leading to “data silos” (Giest, 2017).

## Empirical setting: utilizing data from sensors in Swiss sewer systems

Smart metering, in the context of urban water management, refers to the installation of sensors and data transmission technologies in sewer systems (Apráez & Lavrijssen, 2019). Such sensors allow obtaining real-time information on the system’s actual performance (leakages, outages, etc.) and its impact on surface water quality, thus creating benefits for operators, planners and authorities (Fletcher & Deletic, 2007; Langeveld et al., 2013). For operators, the information gained from sensors is key for real-time operational decision-making (e.g., in case of blockages or malfunctioning of pumps) and for a better understanding of the system’s capacity and behavior. For planners, using available long-term data series for model validation and calibration, reduces uncertainties and thus prevents unnecessary investments, leading to an improved infrastructure planning process (Korving & Clemens, 2002). Furthermore, responsible authorities who receive periodical performance reports could potentially use this information for compliance assessment and more evidence-based decision-making regarding both the operational performance and the systems’ efficacy for surface water protection (Lewis, 2015; Pollitt, 2013; Rieckermann et al., 2017). Representatives from Swiss sub-states reported that, for example, sensor data reveals deficits in the operation of the sewer systems, which can be directly addressed with appropriate measures. The successful implementation of measures leads to an improved infrastructure performance, and therefore has a positive impact on the protection of surface waters. Only through evidence from data do the hidden underground sewer systems become visible, making it possible for the actual functioning of the built infrastructure assets to be examined. The availability of such evidence may also prove useful in moving forward in terms of open data practices and the releasing of public sector information (Conradie & Choenni, 2014; Henninger, 2013). Disclosure of information about sewer system performance ensures transparency and can contribute to an increasing awareness of the public.

In Switzerland, where sewer systems are mainly managed publicly, an increasing number of municipalities install sensors without being obliged to do so (Rieckermann et al., 2017). Swiss municipalities—or the wastewater associations that they create—are to a large degree autonomous in their decisions on how to manage their wastewater systems and whether to invest in digital transformation. Partly due to this autonomy, even in cases where data is available, it is currently often not shared with other beneficiaries such as authorities, or not analyzed for evaluating the system’s performance (Manny et al., 2018).

In Switzerland, the overarching goals of quality, security, and resource protection are embedded in the Swiss Constitution and in the national legislation (such as the Water Protection Act). Given the federalist setting of Switzerland (Linder & Vatter, 2001), the regulative and executive competences on water and infrastructure management are mostly at the sub-state level (alongside with other typical sub-state competences such as education or traffic infrastructure). Sub-state authorities are units of public administration of the sub-state and have the responsibility to check whether water protection targets are met as defined by law and directives. Operational competences for the discharge and treatment of wastewater have typically been delegated to municipalities who operate the infrastructure (Luís-Manso, 2005), and—given again the federalist structure of Switzerland and far-reaching financial autonomy of Swiss municipalities—the coercive power of sub-states on municipalities on how to exactly perform their duties is limited (Klaus, 2020; Ladner et al., 2016). In the Swiss federalist system, decisions are based on interactions between the sub-state authorities and municipalities. Furthermore, due to the newness of the issue, no clear responsibility has been established. So far, reporting data from sewer systems is not required by any form of sub-state law or directive, and the evaluation of available data by sub-state authorities is also not legally binding.

Our research design benefits from this federalist structure of Switzerland and compares 23 out of 26 Swiss sub-states (see “Table 4 in Appendix 1”).^2^ Given that Swiss sub-states have important competencies in the domains of wastewater management and digitalization, we can observe different outcomes and related conditions for different sub-states. At the same time, we keep the basic legal and institutional structure of Switzerland constant. For these reasons, comparing Swiss sub-states has been a popular approach (Kammermann, 2018; Klaus, 2020; Thomann, 2015). Furthermore, while the federalist structure might be a specificity of the country, we consider Switzerland as a typical case (Seawright & Gerring, 2008) in terms of challenges related to digitalization of infrastructure sectors, comparable to other (Western, democratic) countries.

## Methods

### Data collection

**Fig. 1:**
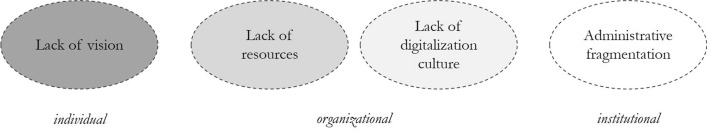
Identified conditions hindering the evaluation of data by Swiss sub-states: Lack of vision at the individual level, lack of resources and lack of digitalization culture at the organizational level and administrative fragmentation at the institutional level

We relied on a two-step approach for data collection. First, we conducted nine exploratory expert interviews (with operators, authorities, engineers, and researchers) in April 2017 that helped us to specify the conditions that may be particularly relevant for our analysis, based on the general model derived from the combination of existing theories. In our study context, we conceptualize these conditions as barriers to digital transformation (see Fig. 1). Our choice on analyzing barriers instead of driving or enabling conditions is partly driven by the collected data revealing that cases with absence of data utilization are overrepresented. Second, we gathered data from semi-structured face-to-face interviews with 23 sub-state representatives (data stems from questionnaires for three sub-states) in October 2017. Sub-states’ representatives are affiliated to the sub-states’ division of urban water management.^3^ The interviews lasted between 1 and 3 hours, and included questions on the infrastructure, sensors, and using data, as well as the representative’s preferences for given policy instruments (see Table 1 for detailed interview questions used in our analysis). The interview data was then used to calibrate the outcome and two conditions as part of applying a Qualitative Comparative Analysis (QCA).

### Qualitative comparative analysis (QCA)

We apply Qualitative Comparative Analysis (QCA) to identify configurations of barriers at different levels, given that we expect conditions from the three levels to interact. QCA is ideally suited for this task, because it identifies different combinations of conditions linked to an outcome (Ragin, 1987; Rihoux & Ragin, 2009; Schneider & Wagemann, 2012). The method, which is based on set-theory and Boolean algebra, is typically utilized for the comparison of a medium (5–50) number of cases. Thus, the number of Swiss sub-states (26, from which we include 23 in our analysis) aligns well with the medium-N focus of QCA (Schneider & Wagemann, 2012). In similar settings, scholars have used QCA to compare Swiss sub-states (Sager & Rielle, 2012), to assess the influence of water stress, geographic and economic conditions on water recycling in Australia (Kunz et al., 2015), or the use of body-worn cameras policy in US states (Pyo, 2020).

Fuzzy-set QCA (fsQCA) relies on data points on an interval scale (from 0 to 1) to identify their degree of membership in the sets of the outcome and the conditions. A so-called truth table is the basis of the analysis, and presents all observed and logically possible configurations of conditions. For each configuration of conditions (corresponding to a row in the truth table), the researcher then assesses the degree to which it is empirically related to the outcome. The assessment of this relation is based on fuzzy-set values of all cases and is indicated by a consistency score. Configurations that are consistently related to the outcome are included in the analysis. QCA then reduces the configuration of conditions that are related to the outcome by eliminating redundant conditions and finally identifies sufficient conditions—i.e., conditions that always lead to the outcome, but that are not the only explanations for the outcome. In this work, the analysis was performed using the R-packages “QCA” (Dusa, 2019) and “SetMethods” (Oana et al., 2018).

### From three levels to four conditions

From the literature, we have deductively identified a general model including three levels—individual, organizational, and institutional levels—important for understanding digital transformation. We now inductively combine this theoretical model with case knowledge and insights from expert interviews in order to specify conditions that are particularly relevant for our analysis. This results in four conditions assigned to the three levels identified based on the theoretical discussion (see Fig. 1).

First, at the individual level, the literature considers attitudes, beliefs, and vision of responsible individuals as critical in creating the impetus for innovation in general (Rogers, 2003), and data-driven management more specifically (Corbett & Webster, 2015). The role of those individuals responsible for the respective administrative divisions was also emphasized as a crucial condition for the evaluation of data in our exploratory interviews. For example, an expert stated that “evaluating data is often rather recognized as a hobby of those skilled to deal with data instead of generally acknowledging it as an important task that may reduce surface water pollution.” Therefore, lack of vision about a future digitalized functioning and evaluation of the water infrastructure sector on the part of individuals such as division or department heads in sub-state authorities is considered a potential barrier for the evaluation of data from sewer systems.

Second, as suggested by the literature, a lack of personnel resources and capacities at the organizational level (Klievink et al., 2016) stands out as another potentially impeding condition. For example, an expert explained that “evaluating data requires additional administrative efforts.” In line with the literature, findings from exploratory expert interviews indicate that personnel capacities are often insufficient, which may explain why authorities get into difficulties concerning time and workload management when it comes to regular evaluations of data.

Third, again on the organizational level, to complement quantitative aspects of available resources at the organizational level, we include a more qualitative condition, i.e. the digitalization culture in the sub-state, as a further condition influencing data evaluation. As suggested by both the literature as well as our exploratory interviews, an appropriate innovation culture is essential for successful initiatives of big data utilization (Marshall et al., 2015; Surbakti et al., 2019).

Fourth, on the institutional level, our insights from exploratory expert interviews suggest that one of the most important condition is institutional fragmentation. The higher the numbers of municipalities that a sub-state authority has to deal with, the higher the level of institutional or administrative fragmentation. Such higher fragmentation increases transaction costs of collaboration among the concerned organizations (Lubell et al., 2017). In our case, we argue that the higher the fragmentation, the more costly it is to exchange and jointly evaluate data among local municipalities and sub-state authorities. We rely on this specific condition in order to represent how institutional aspects can create barriers to innovation and digital transformation. In Switzerland, the operation of the majority of sewer systems lies in the hand of 2′222 municipalities (BFS, 2018). However, sub-states differ strongly in terms of number of municipalities within their jurisdictions. In terms of data evaluation, sub-state authorities may be hindered by huge administrative burdens, as many municipalities imply many sources of data that need to be evaluated.

Along with our general theoretical model, we expect the four conditions at the individual, organizational, and institutional levels to interact and potentially jointly act as barriers to the utilization of data in Switzerland’s urban water management practice. For example, it could be that the lack of vision at the individual level represents a barrier to digital innovation only if there is a simultaneous lack of digitalization culture. Or, institutional fragmentation might be a barrier only if resources at the organizational level are lacking. Also, it could be that there are several alternative sufficient conditions to a lack of digital transformation, pointing toward an equifinal solution, i.e., one aspect of configurational complexity emphasized by QCA (Ragin, 1987). Whereas these examples illustrate the potential presence of configurational complexity, and thus justify the reliance on QCA, we have no strong configurational expectations and basically follow an abductive logic (Aliseda, 2006a, 2006b; Fischer & Maggetti, 2017): We have directional expectations for the influence of each condition on the utilization of data in Switzerland’s urban water management practice, but identify configurations of conditions based on the inductive nature of QCA.

### Operationalization and calibration

The following presentation of the calibration procedure—that is, the assignment of fuzzy-set values to different states of the outcome and conditions—includes a discussion of the documentary sources for operationalizing each condition, as well as a discussion of our choices on fuzzy-set anchors for the outcome and each of the four conditions. Fuzzy-set anchors are based on the researchers justified decisions and were not discussed with experts or interviewees. “Table 5 and 6 in Appendix 1” provide an overview of the raw and calibrated data. At the level of data used for assessing the four conditions and outcomes describing the cases of sub-states, we rely on information from different sources. For example, the condition situated at the individual level is calibrated based on the perception of the individual representative in the sub-state authority drawn from the semi-structured interviews in 2017. The data describing the sub-state authority is used for the calibration of conditions at organizational level and the institutional level is represented by external data sources (BFS, 2018; Schmid et al., 2018) describing the entire sub-state in the year 2018. The outcome is defined according to the indications of the individual representative within the respective sub-state authority. Therefore, our unit of analysis is the sub-state, but we rely on information from smaller units within that sub-state whenever that is appropriate for operationalizing the conditions. Consequently, we incorporate multiple levels of measurement, while the outcome of our analysis addresses sub-state authorities.

#### Outcome: no data evaluation (NO-EVA)

We describe the outcome by whether sub-state authorities do not evaluate data on the performance of sewer systems (NO-EVA). Due to the asymmetric distribution of the outcome, with more cases present where data is not evaluated, we focus on the absence of what we assume are “driving or enabling conditions” (i.e. barriers). In this sense, we can study how barriers could be approached to improve current practices.

The outcome NO-EVA is measured by an interval-scaled variable which is associated to the multiple-choice interview question: “How often is monitoring data evaluated by the sub-state authority?” Fuzzy-set calibration is then based on respective answers. A full set membership score (1.0) is assigned to cases where data is not evaluated. A full set non-membership score (0.0) is given to cases where sub-states’ representatives evaluate data after relevant surface water pollution incidents (e.g., heavy rainfalls) and additionally in a periodic scheme, either monthly or yearly. Generally, both frequencies, i.e., monthly or yearly, would be a comparatively satisfactory state of practice and would provide useful evidence on the system’s performance. We assume that evaluating data at least after relevant pollution incidents is already a step toward evidence-based management and thus assign a fuzzy-set score of 0.67, which is, for instance, the case in FR. The cross-over point of 0.5 is set for the shift from an incident-based toward a periodic data evaluation. If data evaluation solely occurs periodically without incident-based evaluation, we assign a fuzzy-set score of 0.33.

#### Condition 1: lack of vision (LACKVIS)

The condition *lack of vision* (LACKVIS) is calibrated based on an ordinal variable that reflects the openness of the sub-states’ representative toward a specific future scenario of “data-driven management of (smart) sewer systems.” The condition does thus not assess whether the sub-states have any vision for the future, but whether they have a specific vision that we assume would help them to work in the direction of relying on digitalization trends for data evaluation. The following scenario was presented to the interviewee: “[…] [the] sewer system [is] equipped with multiple sensors. Data is continuously transferred live and wireless to a central control system, automatically checked and then archived. Authorities receive automatically generated reports that they use a) to evaluate the functioning of the system, b) to make evidence-based decisions and c) to develop and implement necessary measures with the objective of meeting water protection targets.” Full membership (1.0) for the set LACKVIS is assigned if the interviewee disagrees with the outlined scenario, full set non-membership (0.0) is given to a full scenario acceptance. An additional set membership score of 0.4 is introduced to consider if interviewees (e.g., FR, NE) agree only under specific preconditions, e.g., if competences and responsibilities are to be distributed more clearly. We assume that such a situation lies more within the set of agreement than disagreement, which is why a score of 0.4 is assigned. Some sub-state representatives (e.g., SO, TG) welcome data-based management of sewer systems. On the contrary, other representatives (e.g., GR, OW) argue that sensors in the challenging sewer environment are not yet state of the art technology and are rather convinced that either the technology is not leading to any form of improvement, or that the vision is impossible to ever materialize. Hence, such cases are assigned a score of 1.0.

#### Condition 2: lack of resources (LACKRES)

For the measurement and calibration of the condition *lack of resources* (LACKRES), we consider personnel capacities within the responsible division of urban water management of the sub-state authority. We rely on personnel capacities rather than financial resources due to limited data availability for the latter.^4^

**Fig. 4:**
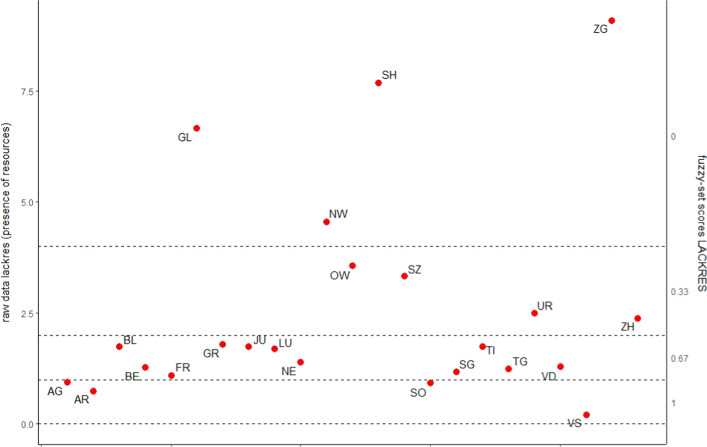
Condition LACKRES

However, we calculate the personnel capacities of each sub-state authority controlling for number of municipalities in each sub-state. This allows us to consider scale effects related to the different number of municipalities per sub-state, and thus the different number of potential interactions for sub-state authorities when dealing with data evaluation. Similar to the calibration procedure used by Kammermann (2018), we assume that by dividing the percentage of full-time job positions by the number of municipalities in a sub-state, we can provide a relative estimate of organizational capacities (OC). For instance, sub-states with many municipalities (e.g., BE (351 municipalities), ZH (168)) generally require more employees at the sub-state level to fulfill the same tasks than sub-states with fewer municipalities (e.g., GL (3), NW (11)). The cross-over point of 0.5 is fixed for an OC ratio of 2.0, which is chosen based on the following consideration. A value of 0.5 could, for instance, be generated if a case features a 100 percent full-time position who is responsible for 50 municipalities, solely in terms of urban water management. Set membership scores for LACKRES are assigned in the following scheme: full set non-membership (0.0) for an OC ratio > 4, full set membership (1.0) for an OC ratio < 1. Assigning these fuzzy-set scores is based on a combination of visual inspection of the raw data distribution (see “Fig. 4 in Appendix 1”) and logical considerations on anchors as mentioned above. Therefore, our calibration procedure is characterized by the fact that it does not rely solely on mathematical measures such as mean or deciles.

#### Condition 3: lack of digitalization culture (LACKDIGI)

**Fig. 5:**
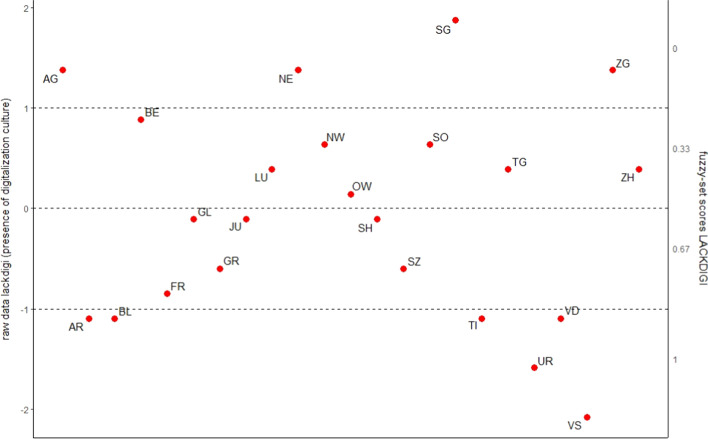
Condition LACKDIGI

To operationalize the condition of a *lack of digitalization culture* (LACKDIGI) in the sub-state authority, we rely on a digitization index as calculated by Schmid et al. (2018) for Switzerland’s sub-state authorities. The index includes variables on e-governance, e-voting and specific online services (Schmid et al., 2018). Thereby, it describes how engaged the sub-state authority is in adopting digital innovations within their service provision and indicates the general attitude toward digitalization among public actors. The measurement of the condition LACKDIGI corresponds to Schmid et al. (2018) digitization index which varies between values of 1.88 and − 2.08. In the process of fuzzy-set calibration, a cross-over point at value 0 is defined as the mean of the values corresponds to − 0.04 (see “Fig. 5 in Appendix 1”). Full set non-membership score (0.0) is assigned to digitization index values > 1, full set membership score (1.0) to values < − 1. Further choices for fuzzy-set anchors are derived from the distribution of index values.

#### Condition 4: administrative fragmentation (FRAG)

The condition a*dministrative fragmentation* (FRAG) is operationalized based on the number of municipalities *N*_mun_ in a given sub-state. With respect to the variable distribution, we employ fuzzy-set scores in the following logic. A sub-state with a comparatively small *N*_mun_ is regarded as highly centralized in its administration, and hence not fragmented at all. A full set non-membership score (0.0) is therefore assigned to *N*_mun_ ≤ 25. In contrast, a sub-state with many municipalities is presumably strongly fragmented regarding its administration. Thus, the full set membership score (1.0) is given to *N*_mun_ > 250. In the sub-state BE, the maximum number of municipalities (*N*_mun, max_ = 351) is reached. The cross-over point (0.5) is chosen for *N*_mun_ = 60, based on the variables’ distribution. Further differentiating fuzzy-set scores can be found in Table 1.

**Table 1:** Calibration of outcome and conditions

	Explanation	Measurement	Operationalization
NO-EVA	Frequency of data evaluation on the performance of sewer systems by the division of urban water management in a sub-state authority	Ordinal variable: “How often is monitoring data evaluated by the sub-state authority?” 0: no data evaluation 1: incident-based data evaluation 2: periodic data evaluation 3: incident-based and periodic data evaluation	Manual fuzzy-set membership scores: 0: periodic data evaluation and after relevant incident 0.33: periodic data evaluation 0.67: incident-based data evaluation 1: no data evaluation
LACKVIS	Lack of vision of the individual representative in the division of a sub-state authority	Ordinal variable: “Is the sub-state representative agreeing to a possible future digital scenario?” 1: Agreement to vision 0.5: Agreement under precondition 0: Disagreement to vision	Manual fuzzy-set membership scores: 0: agreement to vision 0.4: agreement under preconditions 1: disagreement to vision
LACKRES	Lack of (human) resources in the division of a sub-state authority	Organizational capacities (${\text{OC}}$) are measured by the percentage of full-time jobs [%] in the authority’s division of urban water management divided by the number of municipalities in each sub-state ${\text{OC}} = \frac{ {\text{Full-time jobs [\%]}} }{N_{\text{mun}}}$	Manual fuzzy-set membership scores: 0: ${\text{OC}} > 4$ 0.33: $2 < {\text{OC}} \le 4$ 0.67:$1 \le {\text{OC}} \le 2$ 1:${\text{OC}} < 1$
LACKDIGI	Lack of digitalization culture in a sub-state authority	Schmid et al. (2018) provide an index on the digitization of Swiss sub-state authorities $D_{{{\text{index}}}}$	0.33: $0 < D_{{{\text{index}}}} \le 1$ 0.67: $- 1 < D_{{{\text{index}}}} \le 0$ 1:$D_{{{\text{index}}}} \le - 1$
FRAG	Administrative fragmentation in a sub-state	Administrative fragmentation is measured by the number of municipalities $N_{{{\text{mun}}}}$ in a sub-state	Manual fuzzy-set membership scores: 0: $N_{{{\text{mun}}}} \le 25$ 0.2:$25 < N_{{{\text{mun}}}} \le 50$ 0.4:$50 < N_{{{\text{mun}}}} \le 75$ 0.6:$75 < N_{{{\text{mun}}}} \le 150$ 0.8:$150 < N_{{{\text{mun}}}} \le 250$ 1:$N_{{{\text{mun}}}} > 250$

## Analysis and results

Table 2 presents the truth table for the outcome NO-EVA, that is, the fact that sub-states do not evaluate data on the performance of sewer systems. The combination of four conditions results in 16 possible configurations, from which 12 are empirically observed in the dataset and appear in Table 2 (that is, 12 configurations have empirically observed cases that are strong members in the respective set). This leaves us with four logical remainders that are not displayed in the truth table.

**Table 2:** Truth table for the analysis of sufficiency for NO-EVA

LACKVIS	LACKRES	LACKDIGI	FRAG	NO-EVA	Consistency	PRI	Cases
0	1	0	1	1	1.00	1.00	BE, LU, SO, TG
0	1	0	0	1	1.00	1.00	NE
1	0	0	0	1	1.00	1.00	OW
1	1	1	0	1	1.00	1.00	AR
1	1	1	1	1	1.00	1.00	GR
1	0	1	0	1	0.98	0.97	UR
0	1	1	0	1	0.98	0.96	JU
1	1	0	1	1	0.96	0.94	AG, SG
0	1	1	1	1	0.95	0.91	BL, FR, TI, VD, VS
0	0	0	1	0	0.88	0.74	ZH
0	0	1	0	0	0.86	0.67	GL, SH, SZ
0	0	0	0	0	0.70	0.33	NW, ZG

*PRI* proportional reduction in inconsistency

Raw consistency threshold: **0.9**

We set the consistency threshold for configurations leading to the outcome at 0.9, a choice that is based on two complementary criteria. First, this threshold corresponds to a major gap in consistency and PRI scores. A high consistency score of a configuration indicates sufficiency of that configuration for the outcome (see also “Appendix 2”). Second, this threshold clearly separates configurations covering cases that show the outcome from configurations covering cases that do not show the outcome. Two exceptions are SH and SZ, covered by a configuration non-sufficient for the outcome even though being a strong member in the outcome, and VS, covered by a configuration sufficient for the outcome even though not being a member in the set of cases with the outcome. An alternative threshold of 0.8 would be lower but still acceptable in terms of consistency, but the related result (LACKVIS + LACKRES + LACKDIGI + FRAG) would include more cases (three instead of one) that are only weak members in the outcome set (see also “Appendix 4”).

The first nine configurations thus all lead to the outcome NO-EVA, whereas the empirical evidence that the latter three also lead to such an outcome is not consistent enough. The last truth table column lists the empirical cases mainly covered by each configuration. Given that the analysis focuses on barriers, we only present and discuss results for the outcome NO-EVA, and refer to “Appendix 2” for a presentation of results for sub-states where data is actually evaluated (no-eva^5^). For NO-EVA, the analysis of necessity reveals that there is no individually necessary condition (see “Table 7 in Appendix 2”).

The configurational information in the truth table is then subjected to the Quine–McCluskey algorithm, which returns different logical solution terms that vary in their complexity, depending on how they take configurations without information on the consistency for the outcome into account (Schneider & Wagemann, 2012). We restrict the presentation of results to parsimonious solution (see Table 3). The parsimonious solution is equal to the intermediate solution,^6^ the conservative solutions appear in “Table 11 in Appendix 2.”

**Table 3:** Parsimonious solution

Solution term	LACKVIS	+	LACKRES	NO-EVA
Single case coverage	OW, UR; AG, SG; AR, GR		NE; BE, LU, SO, TG; JU; BL, FR, TI, VD, VS; AG, SG; AR; GR	
Consistency	0.96		0.92	
Raw coverage	0.58		0.70	
Unique coverage	0.17		0.29	
Solution consistency				0.93
Solution coverage				0.87

The raw consistency threshold was set at 0.9

The solution consists of two alternative individual conditions sufficient for the non-evaluation of data.^7^ The consistency and coverage scores express to what extent statements about set-theoretic relations between conditions and the outcome are supported by empirical evidence. An overall consistency score of 0.93 indicates that the solution is consistent with empirical evidence from the cases to a very large degree. An overall coverage value of 0.87 means that 87% of the outcome values are covered by the solution formula. Four out of 17 cases are covered by more than one of the two conditions (AG, SG, AR, GR). The case labels appear in the table together with each solution. Robustness of a) the fuzzy-set values to the exact calibration procedure and b) results to changes in the calibration procedure and fuzzy-set values are discussed in “Appendix 4.” The robustness tests revealed that our results are robust with respect to the calibrations of LACKVIS, LACKRES, and FRAG and more sensitive to the calibration of LACKDIGI (s. “Appendix 4”).

The findings reveal an equifinal solution, i.e., a situation where two alternative conditions can cause the outcome. They show that data is not evaluated in sub-states where individual representatives of the sub-state authority are lacking a vision (first term), or where the division of urban water management in the sub-state authority are lacking resources (second term). In the following, we interpret each solution through the lenses of case knowledge and insights, in order to complement and validate the results of the comparative analysis.

A first solution term for why sub-states do not evaluate data is given by the condition of a lack of vision at the individual level (LACKVIS) and covers six sub-states. Half of the six sub-states covered by this solution, OW, UR, AR, are some of the smallest sub-states of Switzerland by size and population. Indeed, digital transformation can have more relevance and value for larger sewer networks, as stated by the sub-state representative of AR. Size could thus partially correlate with a lack of vision at the individual level, potentially due to reduced professionalism and missing awareness for the necessity of evaluating data. However, according to the configurational nature of our approach, this is not the only important condition, given that another small sub-state (NW) is actually the most progressive regarding evaluating data from sewer systems. While the representative of the sub-state ZG claims that “the vision is already in realization, i.e., steps are undertaken to realize the presented future scenario,” the representative of the sub-state UR rejects the scenario on the grounds that “it is not the authority’s responsibility to evaluate data.”

The second solution term (LACKRES) demonstrates that in most sub-state authorities (15 out of 18), personnel resources are generally limited and therefore may be lacking for the novel task of evaluating data from sewer systems. Thus, even though a vision for digital transformation of urban water management may be present, evaluation of data fails due to the unavailability of sufficient organizational capacities, i.e. human resources, at the level of the sub-state authority. For instance, the sub-state SO is generally very innovative and one of the most motivated sub-states in Switzerland when it comes to digitalization of urban water management. Since 2017, SO provides an online database where information on specific infrastructure elements is stored and openly available. In addition, together with the sub-state BE, SO created a digital data management concept to build up necessary IT infrastructures for a smarter management of sewer systems in the future. However, even though innovativeness and vision are clearly present, the sub-states SO and BE do not have sufficient personnel to evaluate data from sewer systems on a regular basis. In the sub-state TG, the situation is similar: though their openness toward digitalization is comparatively high (e.g., they conducted a survey on sewer data management among their municipalities in 2017), organizational capacities at the authority’s level are lacking. In the sub-state GR, the division representative also expressed his concern about the lack of human resources. Finally, the representative from the sub-state TG emphasized a different aspect of human resources, saying that “it is not so much about capacities in numbers, but rather that the technical expertise of the personnel is currently inadequate.”

The analysis reveals the sub-state VS as a contradictory case, in the sense that the sufficient condition LACKRES is present, but not the outcome NO-EVA. In fact, the sub-state VS represents a configuration where data are evaluated, but resources are lacking. Given our context knowledge, we assume that the individual representative of the sub-state VS has a comparatively strong vision toward data evaluation (lackvis) which may dominate the absence of resources (LACKRES) in this case. The individual sub-state representative has strong interests in water protection and therefore may act as a championing figure with high personal commitment (Surbakti et al., 2019) even though human resources are lacking.

Both conditions LACKDIGI and FRAG are not part of the solution as shown in Table 3. Therefore, the absence of data evaluation (NO-EVA) cannot be attributed to a lack of digitalization culture at the organizational level or administrative fragmentation at the institutional level. The additional analysis of the negated outcome, that is, the evaluation of data (see “Table 10 in Appendix 2”), reveals that authorities having a vision at the individual level (lackvis), having sufficient resources at the organizational level (lackres) and not being confronted with administrative fragmentation (frag) evaluate data.

## Discussion

Based on the literature on digital transformation (Mergel et al., 2019), big data utilization (Apráez & Lavrijssen, 2019; Giest, 2017; Klievink et al., 2016; Maciejewski, 2016) and organizational innovation (Mergel et al., 2016; Sun et al., 2016) as well as case knowledge and insights from expert interviews—thereby systematically combining deductive and inductive logics—we have tested the joint influence of four different conditions as hindering data evaluation by public authorities. Potential barriers in our model stem from individual (Corbett & Webster, 2015; Rogers, 2003), organizational (Klievink et al., 2016; Marshall et al., 2015; Surbakti et al., 2019) and institutional (Lubell et al., 2017) levels, in line with literatures on technological innovation (Tornatzky et al., 1990) or public administration (Scharpf, 2018). Results show that conditions at two levels affect data utilization in alignment with our expectations—the presence of a lack of vision (LACKVIS) or a lack of resources (LACKRES) hinders data evaluation, pointing toward an equifinal solution, with two alternative individually sufficient conditions leading to a lack of data evaluation.

The barrier with the highest coverage value, which also acts as an explanation for 15 out of 18 cases where data is not evaluated—a lack of resources at the sub-state level—might comparatively be easy to change for sub-state authorities. Of course, simply providing more financial resources to either sub-state authorities or municipalities might not help, but additional financial resources could help establishing relevant network, education or learning (Bennett & Howlett, 1992) opportunities across and within sub-states and municipalities. These opportunities could also address the barrier of a lack of vision at the individual level. Furthermore, additional resources could be spent to specifically hire personnel with visions related to furthering digitalization and related data evaluation.

The conditions present in the solution term are also linked with other conditions across levels of our theoretical model. This is not a problem from a methodological point of view, given that QCA views cases as configurations of conditions (Mahoney, 2004; Ragin, 1987), and correlations between these conditions are moderate only.^8^ Yet, it could be argued that the absence of digitalization culture (LACKDIGI) at the organizational level may influence a lack of vision (LACKVIS) at the individual level, a mechanism that should be tested in further studies. Our cases however show that lack of vision (LACKVIS) might be absent also in the presence of a digitalization culture (lackdigi) (e.g., BL, VS). Additionally, LACKRES and FRAG are related, as both include the number of municipalities in a given sub-state. However, only one condition appears in the solution term (LACKRES), meaning that LACKRES serves as a sufficient representation for cases with the outcome NO-EVA, whereas FRAG does not.

Among the four conditions, two conditions, i.e. the general digitalization environment (LACKDIGI) and administrative fragmentation (FRAG), are not sufficient conditions for data evaluation to happen. With respect to LACKDIGI, it could be that digitalization in the wastewater sector is not directly related to digitalization in other departments or sectors. Public authorities are silo-structured (Bouckaert et al., 2016), and some departments may be more advanced than others. The indicator by Schmid et al. (2018) refers to very general aspects, e.g., e-government or digital services, and might not represent particular departments within sub-state authorities and how they deal with novel digital solutions. With respect to FRAG, our results suggest reducing institutional fragmentation by municipal mergers (Steiner, 2003) or the creation of wastewater associations among multiple municipalities (Hulst & Van Montfort, 2007) per se—i.e., without an increase in relevant resources—might not be helpful to foster digitalization. Yet, if we apply a lower consistency threshold in the truth table (including 4 additional cases, from which only two are strong members in the outcome),^9^ also FRAG and LACKDIGI are individually sufficient conditions for the lack of data evaluation (see “Appendix 4”).

Following previous studies have also identified several conditions from different levels to be important (Sun et al., 2016; Surbakti et al., 2019; Tornatzky et al., 1990), and based on a mix of deductive and inductive logics, that is, theoretical insights and in-depth case knowledge, we identify barriers to digital transformation at different levels. However, there may be other conditions potentially influencing the evaluation of data. For example, besides the vision of the individual responsible for data evaluation at the sub-state authority, the actual data science expertise of the sub-states’ representatives might matter (Klievink et al., 2016). Taking organizational resources into account, we incorporated this condition to some degree—assuming that personal skills can be acquired through financial and personnel resources. However, data science and IT expertise remains an important condition that deserves more scientific attention in future studies.

Furthermore, we have analyzed public actors at the level of sub-states. Sub-state authorities can install strong incentive structures and provide knowledge, but data evaluation is at the end a joint task of sub-states, municipalities and other actors, such as engineering companies. Disentangling the network structures (Fischer, 2017) among the different actors concerned by the challenge of digital transformation—and suggesting how exactly additional resources could foster network interactions—is another task for further studies. Studying the municipalities that are embedded within the sub-states in a hierarchical logic could also be done relying on combinations of QCA and hierarchical linear modeling (Meuer & Rupietta, 2017). Also, other potentially important actors are other political entities acting as role models and creating mimetic pressure (DiMaggio & Powell, 1983), as argued by the literature on innovation diffusion (Rogers, 2003; Shipan & Volden, 2008) or learning (Bennett & Howlett, 1992). Individual case studies of selected cases could help in disentangling the more detailed mechanisms and processes over time that are responsible for why sub-states do or do not evaluate data. By comparing Swiss sub-state authorities to similar bodies, e.g., Water Boards in the Netherlands, Water Authorities in France, or Environmental Ministries of German Länder, which all demonstrate differences in terms of executive, regulative and operative competences, novel explanations to the implementation of data evaluation may be elucidated, and the transferability of our results can be assessed.

## Conclusion

The digital transformation of infrastructure sectors has many potential benefits, especially in urban water management, where only little is known about the performance of sewer systems (Fletcher & Deletic, 2007). However, exploiting the full value of data is hindered by different barriers (Hoppe et al., 2019). This article focuses on digital transformation in the public sector, by studying the barriers to data utilization related to the performance of urban wastewater infrastructure in Swiss sub-states. Results from our fuzzy-set QCA reveal that two out of four conditions—namely lack of vision and lack of resources—indicate why data is not evaluated by sub-state authorities. We thus find evidence of hindering conditions at individual and organizational levels. The QCA method, focusing on causal complexity, has allowed us to identify two equifinal solution terms. Such equifinality is one aspect of configurational complexity (Ragin, 1987). Results are robust in terms of frequency of cases linked to the configurations, as there are several cases for each solution term (Skaaning, 2011). Calibration is grounded on theoretical considerations, case-specific knowledge and the distribution of raw data values. With some exceptions, results are robust to alternative choices of thresholds for conditions and outcomes; the related information appears in “Appendix 4.”

Our analysis contributes to the study of digitalization in organizations by elucidating barriers to utilizing data. The focus on barriers is primarily data-driven, but also allows us to study how deficits could be approached to improve current practices. In Switzerland, the evaluation of data by sub-state authorities is currently fully self-motivated, i.e., there is no coercive pressure (DiMaggio & Powell, 1983) from the institutional context through national-level policies. In the absence of coercive pressure, normative pressure (DiMaggio & Powell, 1983) could also play a role if the current state is suddenly perceived as insufficient (e.g., the lack of digitalization in relations between the national level and sub-states in the health sector has been heavily criticized during the COVID-19 pandemic).

Based on collected data, we explain a specific outcome—why sub-states do not evaluate data from sewer systems. This is an important outcome, as digitalization per se does often not provide immediate benefits, but rather produces large amounts of data leading to data wasting (Mergel et al., 2016), if not shared, treated, and analyzed appropriately (Sun et al., 2016). Conditions favoring or hindering the effective utilization of data have been overlooked in the literature (Maciejewski, 2016; Sun et al., 2016; Surbakti et al., 2019). Our results could also indicate that the utilization of data for evaluation depends on factors on the individual (lack of vision) and organizational levels (lack of resources) directly relevant for hands-on implementation, rather than on more higher-level factors such as institutional fragmentation or digitalization of the public administration in a sub-state.

In terms of transferability, our results are applicable to the sector of urban water management in Switzerland. Yet, we think that our case is also a rather typical case (Seawright & Gerring, 2008) for studying innovation and digitalization in other infrastructure sectors, also in other (rather Western, democratic) countries that face similar challenges. The theoretical model that consists of individual, organizational and institutional conditions is certainly useful for studying different settings and contexts related to innovation and data evaluation practices also in other sectors and countries, and our study should be seen as an effort of theory development in that regard (George & Bennett, 2005). Yet, with respect to transferring the model to other countries, a study of sub-state authorities might be less relevant in non-federalist systems where digitalization and data evaluation might be more strongly steered by the central state.

Our results also speak to discussions about accountability, as data evaluation and the publication of evaluation results can increase transparency and, thus, the accountability of public authorities responsible for sewer systems. In the long term, utilizing data may also go in hand with higher efficiency, both in terms of economic efficiency (i.e., by preventing unnecessary investments due to more available evidence on the systems’ performance) as well as environmental efficiency (i.e. better surface water protection through performance management).

## Figures and Tables

**Fig. 2:**
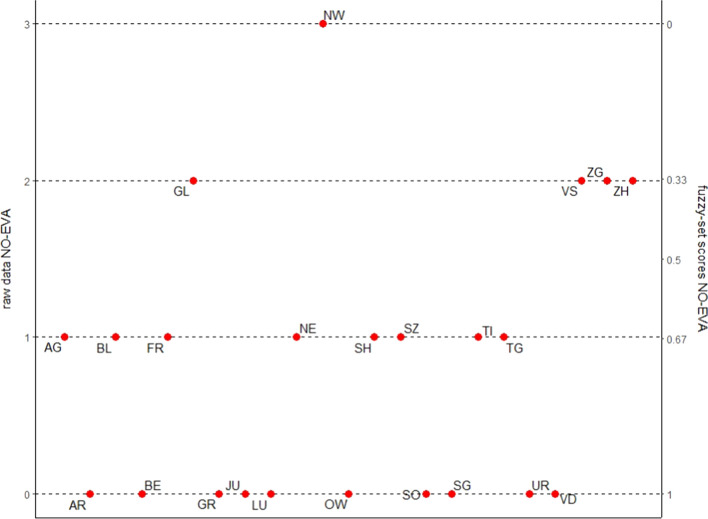
Outcome NO-EVA

**Fig. 3:**
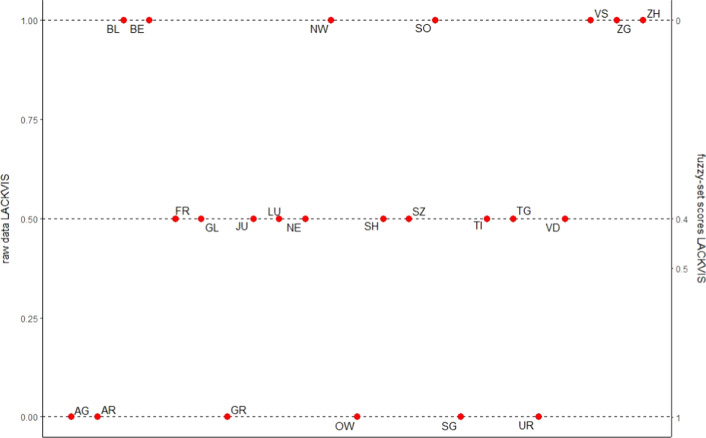
Condition LACKVIS

**Fig. 6:**
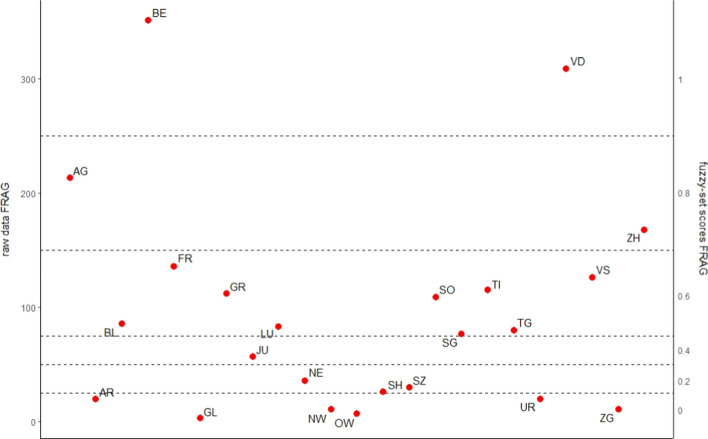
Condition FRAG

**Table 11:**
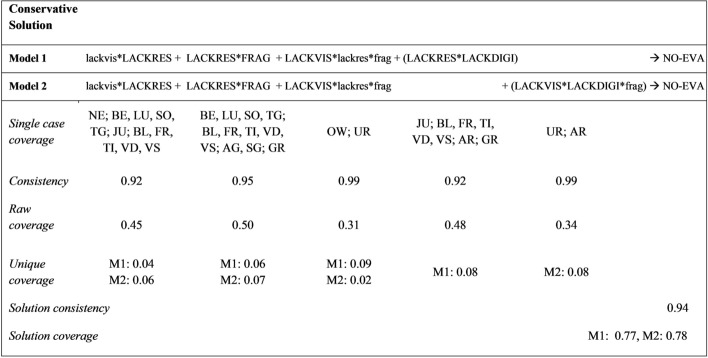
Conservative solution for the occurrence of the outcome (NO-EVA)

The raw consistency threshold was set at 0.9. Number of multiple-covered cases is 13

**Fig. 7:**
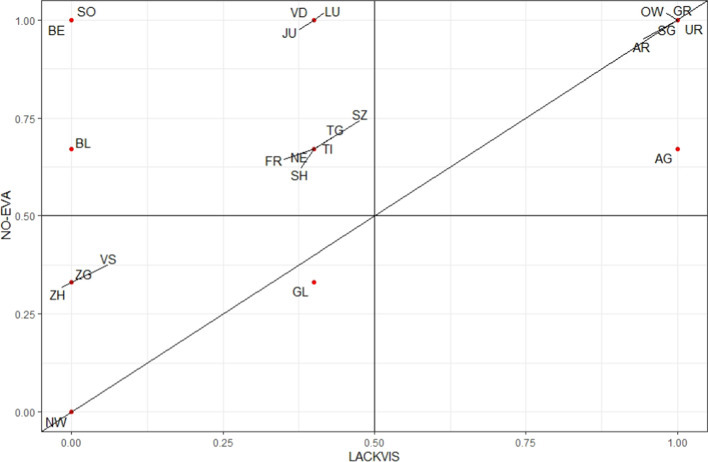
Condition LACKVIS and outcome NO-EVA

**Fig. 8:**
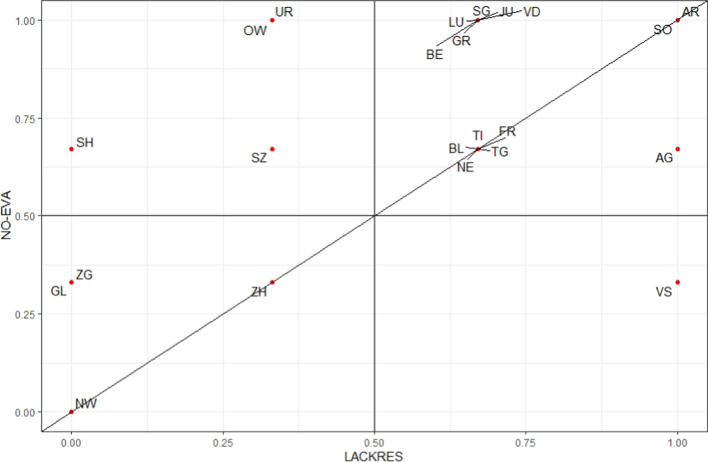
Condition LACKRES and outcome NO-EVA

**Fig. 9:**
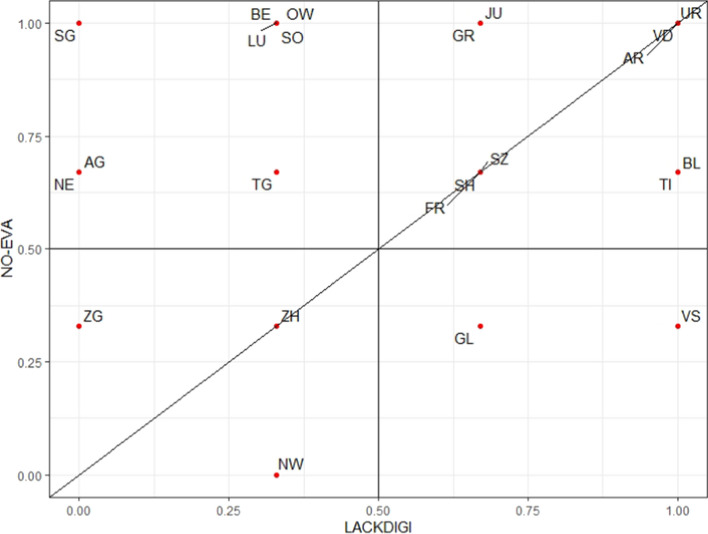
Condition LACKDIGI and outcome NO-EVA

**Fig. 10:**
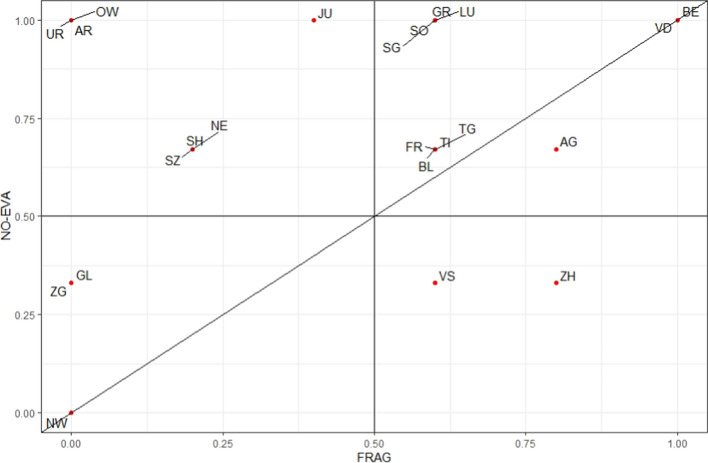
Condition FRAG and outcome NO-EVA

## Data Availability

All own data is included in the manuscript and appendix. All external data is referenced.

## Appendix 1: Sub-states of Switzerland, raw data and fuzzy-set scores

See Tables

**Table 4:** Sub-states of Switzerland and their abbreviations (case IDs)

	Name of sub-state
AG	Argovia
AI*	Appenzell Inner-Rhodes
AR	Appenzell Outer-Rhodes
BE	Berne
BL	Basle-Country
BS*	Basle-City
FR	Fribourg
GE*	Geneva
GL	Glarus
GR	Grisons
JU	Jura
LU	Lucerne
NE	Neuchâtel
NW	Nidwald
OW	Obwald
SG	St. Gall
SH	Schaffhouse
SO	Solothurn
SZ	Schwyz
TG	Thurgovia
TI	Ticino
UR	Uri
VD	Vaud
VS	Valais
ZG	Zoug
ZH	Zurich

*We exclude sub-states AI, BS and GE from the analysis as in these sub-states, the authority itself operates most parts of the sewer system

4,

**Table 5:** Raw data matrix

Case	Outcome: NO-EVA *Data evaluation*	Condition: LACKVIS *Vision*	Condition: LACKRES ${\text{OC}}$	Condition: LACKDIGI $D_{{{\text{index}}}}$	Condition: FRAG $N_{{{\text{mun}}}}$
AG	1	0	0.94	1.38	213
AR	0	0	0.75	− 1.09	20
BL	1	1	1.74	− 1.09	86
BE	0	1	1.28	0.89	351
FR	1	0.5	1.10	− 0.85	136
GL	2	0.5	6.67	− 0.10	3
GR	0	0	1.79	− 0.60	112
JU	0	0.5	1.75	− 0.10	57
LU	0	0.5	1.69	0.39	83
NE	1	0.5	1.39	1.38	36
NW	3	1	4.55	0.64	11
OW	0	0	3.57	0.14	7
SH	1	0.5	7.69	− 0.10	26
SZ	1	0.5	3.33	− 0.60	30
SO	0	1	0.92	0.64	109
SG	0	0	1.17	1.88	77
TI	1	0.5	1.74	− 1.09	115
TG	1	0.5	1.25	0.39	80
UR	0	0	2.50	− 1.59	20
VD	0	0.5	1.29	− 1.09	309
VS	2	1	0.20	− 2.08	126
ZG	2	1	9.09	1.38	11
ZH	2	1	2.38	0.39	168

Key of case IDs: Abbreviations for sub-states in Switzerland

Data stems from semi-structured interviews in October 2017, BFS (2018) and Schmid et al. (2018)

5 and

**Table 6:** Fuzzy-set scores

Case	Outcome: NO-EVA	Condition: LACKVIS	Condition: LACKRES	Condition: LACKDIGI	Condition: FRAG
AG	0.67	1	1	0	0.8
AR	1	1	1	1	0
BL	0.67	0	0.67	1	0.6
BE	1	0	0.67	0.33	1
FR	0.67	0.4	0.67	0.67	0.6
GL	0.33	0.4	0	0.67	0
GR	1	1	0.67	0.67	0.6
JU	1	0.4	0.67	0.67	0.4
LU	1	0.4	0.67	0.33	0.6
NE	0.67	0.4	0.67	0	0.2
NW	0	0	0	0.33	0
OW	1	1	0.33	0.33	0
SH	0.67	0.4	0	0.67	0.2
SZ	0.67	0.4	0.33	0.67	0.2
SO	1	0	1	0.33	0.6
SG	1	1	0.67	0	0.6
TI	0.67	0.4	0.67	1	0.6
TG	0.67	0.4	0.67	0.33	0.6
UR	1	1	0.33	1	0
VD	1	0.4	0.67	1	1
VS	0.33	0	1	1	0.6
ZG	0.33	0	0	0	0
ZH	0.33	0	0.33	0.33	0.8

Key of case IDs: Abbreviations for sub-states in Switzerland

6.

Fuzzy-set scores are based on the calibration procedure of the individual conditions and the outcome as shown in Table 1.

### Plots on the distribution of raw data values and fuzzy-set scores

In the following figures, the x-axis shows the alphabetical order of cases (AG to ZH), the left y-axis shows the raw data used (as listed in Table 5) and the right y-axis shows the fuzzy-set scores (as listed in Table 6).

See Figs. 2, 3, 4, 5 and 6.

## Appendix 2: Analysis of necessity and solution terms

### Analysis of necessity

The analysis of necessity compares one condition and the outcome in terms of set membership scores. For a condition to be ‘necessary’, its set membership requires to be larger or equal to the set membership of the outcome, across all empirical cases. In the following tables, we provide an overview on resulting parameters for the analysis of necessity that are:

- *Consistency of necessity* states whether the presence of the outcome implies the presence of the condition. The parameter is calculated by $Y_{i} \le X_{i} = \sum \left( {\frac{{\min \left( {X_{i} , Y_{i} } \right)}}{{\sum Y_{i} }}} \right)$.
- *Coverage of necessity* expresses the empirical importance of a condition for explaining the outcome, i.e. measures how much of the outcome is covered by the condition. The parameter is calculated by $Y_{i} \le X_{i} = \sum \left( {\frac{{\min \left( {X_{i} , Y_{i} } \right)}}{{\sum X_{i} }}} \right)$.
- *Relevance of necessity (RoN)* indicates whether a condition is trivial or relevant. The parameter is calculated by $\frac{{\sum \left( {1 - X_{i} } \right)}}{{\sum \left( {1 - \min \left( {X_{i} , Y_{i} } \right)} \right)}}$.

See Tables

**Table 7:** Results of the analysis of necessity for the occurrence of the outcome (NO-EVA)

Condition	Consistency	Coverage	Relevance of necessity (RoN)
LACKVIS	0.58	0.96	0.97
LACKRES	0.70	0.92	0.91
LACKDIGI	0.62	0.84	0.84
FRAG	0.55	0.91	0.94
lackvis	0.56	0.74	0.74
lackres	0.44	0.81	0.81
lackdigi	0.50	0.84	0.84
frag	0.61	0.78	0.78

No individual condition is meeting the thresholds for consistency (0.9), coverage (0.5) and relevance (0.5), thus there is no single necessary condition for the outcome NO-EVA

Note that a condition written in uppercase letters marks its presence, in lowercase letters its absence

7 and

**Table 8:** Results of the analysis of necessity for the non-occurrence of the outcome (no-eva)

Condition	Consistency	Coverage	Relevance of necessity (RoN)
LACKVIS	0.43	0.27	0.64
LACKRES	0.52	0.26	0.52
LACKDIGI	0.63	0.32	0.56
FRAG	0.56	0.35	0.67
lackvis	0.94	0.46	0.59
lackres	**0.84**	**0.52**	**0.72**
lackdigi	0.68	0.41	0.66
frag	0.86	0.42	0.57

Necessary conditions meeting the thresholds for consistency (0.7), coverage (0.5) and relevance (0.5)

Thus, lackres (in bold) is a necessary condition for the non-occurrence of the outcome no-eva

Note that a condition written in uppercase letters marks its presence, in lowercase letters its absence

8.

### Analysis of sufficiency

The analysis of sufficiency follows the analysis of necessity. The heart of the analysis is the so-called ‘truth table’ that we present and explain in detail within the text. For each row in the truth table (a ‘configuration’), the following parameters describe the measures of fit:

- *Consistency* measures to which degree a condition is a subset of the outcome. The parameter is calculated by $X_{i} \le Y_{i} = \sum \left( {\frac{{\min \left( {X_{i} , Y_{i} } \right)}}{{\sum X_{i} }}} \right)$.
- *Proportional Reduction in Inconsistency (PRI)* indicates to which degree a condition is a subset of the occurrence of the outcome rather than a subset of the non-occurrence of the outcome. The parameter helps to identify whether the condition is sensitive to being a subset of the occurrence of the outcome and the non-occurrence thereof. It is calculated by $\frac{{\sum \min \left( {X_{i} , Y_{i} } \right) - (\min \left( {X, Y, 1 - Y} \right)}}{{\sum \min \left( {X_{i} , } \right) - (\min \left( {X, Y, 1 - Y} \right)}}$.
- *Raw coverage* expresses how much of the outcome is covered by each solution path. The parameter is calculated by $X_{i} \le Y_{i} = \sum \left( {\frac{{\min \left( {X_{i} , Y_{i} } \right)}}{{\sum Y_{i} }}} \right)$.
- *Unique coverage* states how much of the outcome is covered by only one specific solution path.
- *Solution consistency* measures to which degree the solution path is sufficient for explaining the outcome.
- *Solution coverage* measures how much of the outcome is covered by the entire solution term.

Configurations that pass specified thresholds are then subjected to the Quine-McCluskey algorithm that returns configurations in three types of solutions:

- *Conservative (or complex) solution* includes only configurations that are empirically observed. No assumptions are made about any logical remainders, that is, logical configurations that are not covered by empirical cases.
- *Intermediate solution* considers logical remainders only if they correspond to the assumptions (‘directional expectations’) as defined by the researcher. In terms of complexity, the solution lies in-between conservative and parsimonious solutions.
- *Parsimonious solution* takes into account assumptions about logical remainders in order to return a solution term with the minimum of conditions (least complex solution).

### Truth table and solution terms

See Tables 9,

**Table 9:** Truth table for the non-occurrence of the outcome (no-eva)

LACKVIS	LACKRES	LACKDIGI	FRAG	no-eva	Consistency	PRI	Cases
0	0	0	0	1	0.78	0.51	NW, ZG
0	0	1	0	1	0.72	0.33	GL, SH, SZ
0	0	0	1	0	0.66	0.26	ZH
0	1	1	0	0	0.61	0.05	JU
0	1	0	0	0	0.51	0.00	NE
1	0	0	0	0	0.50	0.00	OW
1	0	1	0	0	0.49	0.03	UR
0	1	1	1	0	0.47	0.09	BL, FR, TI, VD, VS
1	1	1	1	0	0.39	0.00	GR
1	1	0	1	0	0.39	0.00	AG, SG
0	1	0	1	0	0.36	0.00	BE, LU, SO, TG
1	1	1	0	0	0.31	0.00	AR

*PRI*: proportional reduction in inconsistency

Raw consistency threshold: **0.7**

**Table 10:** Conservative, parsimonious and intermediate solution for the non-occurrence of the outcome (no-eva)

Solution term	lackvis * lackres * frag	no-eva
Single case coverage	NW, ZG; GL, SH, SZ	
Consistency	0.76	
Raw coverage	0.76	
Unique coverage	–	
Solution consistency		**0.76**
Solution coverage		**0.76**

The raw consistency threshold was set at 0.7. Number of multiple-covered cases is 0

^10^ and ^11^.

## Appendix 3: Visual interpretation of the relation between conditions and outcome

The following Figs. 7, 8, 9 and 10 present plots of fuzzy-set scores of each individual condition and the outcome NO-EVA. This form of visualization allows to make additional interpretations to those in “Appendix 2” on necessity and sufficiency.

When taking a look at the upper left quadrant in Fig. 7, we observe many empirical cases where the condition LACKVIS is not present (below the cross-over point of 0.5) yet the outcome NO-EVA occurs (e.g., cases BE, SO, BL, LU, etc.). This is contradictory to our assumption that LACKVIS is necessary for the outcome NO-EVA, which makes the cases in the upper left quadrant so-called deviant cases inconsistent in degree. For the analysis of sufficiency, we consider the lower right quadrant where no case is plotted. NO-EVA is not observed, if LACKVIS is not present. Thus, in terms of sufficiency, we do not have any “deviant case inconsistent in degree.” Only the case AG can be classified as a “deviant case inconsistent in kind,” as the fuzzy-set score of NO-EVA is lower than the one of LACKVIS.

Interpretations for Figs. 8, 9, and 10 can be carried out in the same way as explained above. Figure 8 illustrates that the four cases SH, SZ, OW, UR are “deviant cases inconsistent in degree” when it comes to the necessity of LACKRES for NO-EVA. In terms of sufficiency, the case VS represents such a deviant case. Even though resources are lacking (LACKRES), the sub-state authority evaluates data (no-eva).

Figure 9 shows that several cases are “deviant cases inconsistent in degree” regarding the necessity of LACKDIGI for NO-EVA (SG, BE, OW, LU, SO, AG, NE, TG). In these sub-states, even though data is not evaluated (NO-EVA), a digitalization culture is not lacking (lackdigi) at the organizational level. Regarding sufficiency, the cases GL and VS represent such deviant cases.

In Fig. 10, seven cases are “deviant cases inconsistent in degree” regarding the necessity of FRAG for NO-EVA. In these sub-states, data is not evaluated (NO-EVA) while they are not administratively fragmented (frag). For the analysis of sufficiency, the cases ZH and VS are “deviant cases inconsistent in degree.”

Overall, these findings are in line with the calculated parameters of fit in “Appendix 2.”

## Appendix 4: Robustness tests

Robustness tests serve to assess the sensitivity of QCA results. Skaaning (2011) suggests to focus on three aspects: (1) calibration of fuzzy-set membership scores from raw data, (2) empirical evidence in terms of frequency of cases linked to the configurations, and (3) choice of raw consistency thresholds. Whereas all of our solution configurations are covered by multiple cases (criteria (2)), we elaborate on raw consistency thresholds and calibration of fuzzy-membership scores below.

First, as shortly discussed in the main text, our solution is sensitive to the choice of the consistency threshold. In the text, we give two explanations for our choice of a 0.9 threshold. First, we define the consistency threshold based on the first major gap in consistency scores, which lies between 0.88 and 0.95 (see Table 2: Truth table). Second, our consistency threshold separates well cases that are strong members in the outcome set NO-EVA (membership scores higher than 0.5) from cases that are only weak members in NO-EVA (membership score lower than 0.5). If we lower the consistency threshold to take into account the next major gap (below 0.86), the solution changes to LACKVIS + LACKRES + LACKDIGI + FRAG (parsimonious and intermediate solution consistency: 0.85, coverage 0.94). We also shortly mention this solution in the main text, but mainly interpret the solution based on the higher threshold.

Second, alternative calibrations of outcome and conditions appear in tables below together with short comments. With respect to alternative calibrations of the outcome NO-EVA, the solution terms LACKVIS and LACKRES (our main solution) appear in all solutions achieved by different calibration alternatives. The other two conditions, LACKDIGI or FRAG, do never appear as individually sufficient conditions in any of the solutions produced through the alternative calibrations. This robustness result holds even for varying consistency thresholds (0.9 and 0.8). We conclude that given different calibrations, LACKVIS and LACKRES are consistently appearing in the solution term and therefore the presented parsimonious solution is robust concerning the calibration of the outcome NO-EVA.

See Tables

**Table 12:** Robustness of solution concerning calibration of outcome NO-EVA

	Calibration of NO-EVA			Skewness* of NO-EVA	Consistency threshold	Parsimonious solution
	fs-score	Description	Raw data			
Original calibration	0	Periodic data evaluation and after relevant incident	3	78.26%	0.9	LACKVIS + LACKRES NO-EVA
	0.33	Periodic data evaluation	2			
	0.67	Incident-based data evaluation	1			
	1	No data evaluation	0			
Calibration alternative 1	0	Periodic data evaluation	2, 3	78.26%	0.9	LACKVIS + LACKRES * lackdigi NO-EVA
	0.67	Incident-based data evaluation	1		0.8	LACKVIS + LACKRES NO-EVA
	1	No data evaluation	0			
Calibration alternative 2	0	Periodic data evaluation	2, 3	43.48%	0.9	M1: LACKVIS*LACKRES*LACKDIGI + (LACKVIS*lackres *lackdigi) NO-EVA M2: LACKVIS*LACKRES*LACKDIGI + (LACKVIS*lackdigi*frag) NO-EVA
	0.33	Incident-based data evaluation	1		0.8	LACKVIS + LACKRES*lackdigi NO-EVA
	1	No data evaluation	0			

*Skewness: Cases > 0.5/Total number of cases; if skewness equal 50%, there is “no skewness”

Consistency threshold: varies

12.

Our solution is robust with respect to different types of calibration of the condition LACKVIS.

See Table

**Table 13:** Robustness of solution concerning calibration of condition LACKVIS

	Calibration of LACKVIS			Skewness* of LACKVIS (%)	Parsimonious solution
	fs-score	Description	Raw data		
Original calibration	0	Agreement to vision	1	56.52	LACKVIS + LACKRES NO-EVA
	0.4	Agreement under preconditions	0.5		
	1	Disagreement to vision	0		
Calibration alternative 1	0	Agreement to vision	1	69.57	LACKVIS + LACKRES NO-EVA
	0.6	Agreement under preconditions	0.5		
	1	Disagreement to vision	0		

*Skewness: Cases > 0.5/Total number of cases; if skewness equal 50%, there is “no skewness”

Consistency threshold: **0.9**

^13^.

Our solution is robust with respect to different types of calibration of the condition LACKRES.

See Table

**Table 14:** Robustness of solution concerning calibration of condition LACKRES

	Calibration of LACKVIS		Skewness* of LACKRES (%)	Parsimonious solution
	fs-score	Raw data values		
Original calibration	0	${\text{OC}} > 4$	65.22	LACKVIS + LACKRES NO-EVA
	0.33	$2 < {\text{OC}} \le 4$		
	0.67	$1 \le {\text{OC}} \le 2$		
	1	${\text{OC}} < 1$		
Calibration alternative 1	0	${\text{OC}} > 5$	73.91	LACKVIS + LACKRES NO-EVA
	0.33	$2.5 < {\text{OC}} \le 5$		
	0.67	$1 \le {\text{OC}} \le 2.5$		
	1	${\text{OC}} < 1$		
Calibration alternative 2	0	${\text{OC}} > 4$	73.91	LACKVIS + LACKRES NO-EVA
	0.33	$2.5 < {\text{OC}} \le 4$		
	0.67	$1 \le {\text{OC}} \le 2.5$		
	1	${\text{OC}} < 1$		
Calibration alternative 3	0	${\text{OC}} > 2.8$	65.22	LACKVIS + LACKRES NO-EVA
	0.33	$1.8 < {\text{OC}} \le 2.8$		
	0.67	$0.8 \le {\text{OC}} \le 1.8$		
	1	${\text{OC}} < 0.8$		

*Skewness: Cases > 0.5/Total number of cases; if skewness equal 50%, there is “no skewness”

Consistency threshold: **0.9**

^14^.

Our solution is most sensitive to different types of calibration of the condition LACKDIGI (Table

**Table 15:** Robustness of solution concerning calibration of condition LACKDIGI

	Calibration of LACKDIGI		Skewness* of LACKDIGI (%)	Parsimonious solution
	fs-score	Raw data values		
Original calibration	0	$D_{{{\text{index}}}} > 1$	52.17	LACKVIS + LACKRES NO-EVA
	0.33	$0 < D_{{{\text{index}}}} \le 1$		
	0.67	$- 1 < D_{{{\text{index}}}} \le 0$		
	1	$D_{{{\text{index}}}} \le - 1$		
Calibration alternative 1	0	$D_{{{\text{index}}}} > 1$	52.17	LACKVIS + LACKRES NO-EVA
	0.33	$- 0.1 < D_{{{\text{index}}}} \le 1$		
	0.67	$- 1 < D_{{{\text{index}}}} \le - 0.1$		
	1	$D_{{{\text{index}}}} \le - 1$		
Calibration alternative 2	0	$D_{{{\text{index}}}} > 0.8$	52.17	LACKVIS + LACKRES NO-EVA
	0.33	$- 0.1 < D_{{{\text{index}}}} \le 0.8$		
	0.67	$- 1 < D_{{{\text{index}}}} \le - 0.1$		
	1	$D_{{{\text{index}}}} \le - 1$		
Calibration alternative 3	0	$D_{{{\text{index}}}} > 0.8$	39.13	LACKVIS + LACKRES + LACKDIGI NO-EVA
	0.33	$- 0.2 < D_{{{\text{index}}}} \le 0.8$		
	0.67	$- 1 < D_{{{\text{index}}}} \le - 0.2$		
	1	$D_{{{\text{index}}}} \le - 1$		
Calibration alternative 4	0	$D_{{{\text{index}}}} > 0.8$	39.13	LACKVIS + LACKRES + LACKDIGI + FRAG NO-EVA
	0.33	$- 0.2 < D_{{{\text{index}}}} \le 0.8$		
	0.67	$- 1.2 < D_{{{\text{index}}}} \le - 0.2$		
	1	$D_{{{\text{index}}}} \le - 1.2$		

*Skewness: Cases > 0.5/Total number of cases; if skewness equal 50%, there is “no skewness”

Consistency threshold: **0.9**

$D_{{{\text{index}}}}$ mean: − 0.04 and $D_{{{\text{index}}}}$ median: − 0.1

^15^ below). This can be explained by a change of the cross-over point (here: from 0 to − 0.2) which implies that three cases lie above the cross-over point (GL, JU, SH) with calibration alternatives 3 and 4. For calibration alternatives 3 and 4, more conditions are part of the solution: LACKDIGI (alternative 3) and LACKDIGI and FRAG (alternative 4), respectively. Nevertheless, LACKVIS and LACKRES, i.e., our main solution, is still part of both solutions. Given that our calibration of LACKDIGI is based on a simple index (D_index), we propose to stick to numeric calibration anchors, such as the mean of D_index (here: − 0.04) and median (− 0.1). Both values suggest to follow the original calibration or calibration alternatives 1 and 2, which also show a more even distribution in terms of skewness (52.17%).

Our solution is robust with respect to different types of calibration of the condition FRAG.

See Table

**Table 16:** Robustness of solution concerning calibration of condition FRAG

	Calibration of FRAG		Skewness* of FRAG (%)	Parsimonious solution
	fs-score	Raw data values		
Original calibration	0	$N_{{{\text{mun}}}} \le 25$	56.52	LACKVIS + LACKRES NO-EVA
	0.2	$25 < N_{{{\text{mun}}}} \le 50$		
	0.4	$50 < N_{{{\text{mun}}}} \le 75$		
	0.6	$75 < N_{{{\text{mun}}}} \le 150$		
	0.8	$150 < N_{{{\text{mun}}}} \le 250$		
	1	$N_{{{\text{mun}}}} > 250$		
Calibration alternative 1	0	$N_{{{\text{mun}}}} \le 25$	56.52	LACKVIS + LACKRES NO-EVA
	0.2	$25 < N_{{{\text{mun}}}} \le 50$		
	0.4	$50 < N_{{{\text{mun}}}} \le 75$		
	0.6	$75 < N_{{{\text{mun}}}} \le 100$		
	0.8	$100 < N_{{{\text{mun}}}} \le 200$		
	1	$N_{{{\text{mun}}}} > 200$		
Calibration alternative 2	0	$N_{{{\text{mun}}}} \le 25$	47.83	LACKVIS + LACKRES NO-EVA
	0.2	$25 < N_{{{\text{mun}}}} \le 55$		
	0.4	$55 < N_{{{\text{mun}}}} \le 80$		
	0.6	$80 < N_{{{\text{mun}}}} \le 150$		
	0.8	$150 < N_{{{\text{mun}}}} \le 250$		
	1	$N_{{{\text{mun}}}} > 250$		
Calibration alternative 3	0	$N_{{{\text{mun}}}} \le 25$	39.13	LACKVIS + LACKRES NO-EVA
	0.2	$25 < N_{{{\text{mun}}}} \le 60$		
	0.4	$60 < N_{{{\text{mun}}}} \le 90$		
	0.6	$90 < N_{{{\text{mun}}}} \le 150$		
	0.8	$150 < N_{{{\text{mun}}}} \le 250$		
	1	$N_{{{\text{mun}}}} > 250$		
Calibration alternative 4	0	$N_{{{\text{mun}}}} \le 30$	39.13	LACKVIS + LACKRES NO-EVA
	0.2	$30 < N_{{{\text{mun}}}} \le 60$		
	0.4	$60 < N_{{{\text{mun}}}} \le 90$		
	0.6	$90 < N_{{{\text{mun}}}} \le 150$		
	0.8	$150 < N_{{{\text{mun}}}} \le 250$		
	1	$N_{{{\text{mun}}}} > 250$		
Calibration alternative 5	0	$N_{{{\text{mun}}}} \le 25$	39.13	LACKVIS + LACKRES NO-EVA
	0.2	$25 < N_{{{\text{mun}}}} \le 65$		
	0.4	$65 < N_{{{\text{mun}}}} \le 100$		
	0.6	$100 < N_{{{\text{mun}}}} \le 150$		
	0.8	$150 < N_{{{\text{mun}}}} \le 250$		
	1	$N_{{{\text{mun}}}} > 250$		
Calibration alternative 6	0	$N_{{{\text{mun}}}} \le 20$	26.09	LACKVIS + LACKRES + FRAG NO-EVA
	0.2	$20 < N_{{{\text{mun}}}} \le 60$		
	0.4	$60 < N_{{{\text{mun}}}} \le 120$		
	0.6	$120 < N_{{{\text{mun}}}} \le 180$		
	0.8	$180 < N_{{{\text{mun}}}} \le 240$		
	1	$N_{{{\text{mun}}}} > 240$		
Calibration alternative 7	0	$N_{{{\text{mun}}}} \le 15$	60.87	LACKVIS + LACKRES NO-EVA
	0.2	$15 < N_{{{\text{mun}}}} \le 30$		
	0.4	$30 < N_{{{\text{mun}}}} \le 50$		
	0.6	$50 < N_{{{\text{mun}}}} \le 100$		
	0.8	$100 < N_{{{\text{mun}}}} \le 200$		
	1	$N_{{{\text{mun}}}} > 200$		

*Skewness: Cases > 0.5/Total number of cases; if skewness equal 50%, there is “no skewness”

Consistency threshold: **0.9**

^16^.

## Footnotes

1 In the entire text, the term «data» is used to refer to several, often used terms such as «monitoring data» or «sensor data». Such data is collected by digital technologies (e.g., sensors) which are installed within an infrastructure system. For sewer systems, data on water levels, flows, or discharges to surface waters can be measured. As such data is continuously collected, it reaches large volumes and can therefore also be described as big data.

2 In the sub-states AI, BS and GE, the authority operates most parts of the sewer system. Any operational task, e.g., installation of sensors or data gathering, lies in the responsibility of the authority and not as usual in municipal hands. Consequently, we exclude these sub-states from the analysis.

3 Examples of sub-state divisions responsible for urban water management in Swiss sub-state authorities are Amt für Wasser und Abfall (sub-state BE), Amt für Abfall, Wasser, Energie und Luft (ZH), Office de l'environnement (JU).

4 We have no information about the number of personnel specifically working on digitalization within the respective sub-state division, but consider the general personnel capacities as a proxy indicator for the capacity of a division to deal with issues of digitalization, and innovation more generally. Asking the division representative whether they consider they have enough resources would have been another possibility, but that perception would directly depend on what the representatives want to achieve or imagine themselves achieving and thus depend on their vision, among others. Finally, we do not include personnel at the municipality level nor size of municipalities, given that our unit of analysis are sub-states.

5 Conditions and outcome written in lowercase letters stand for their absence, in uppercase letters for their presence.

6 Directional expectations are LACKVIS NO-EVA; LACKRES NO-EVA; LACKDIGI NO-EVA; FRAG NO-EVA.

7 In the Table, + represents logical ‘or.’.

8 Pearson correlation coefficients for conditions are:

- LACKVIS, LACKDIGI: 0.01.
- LACKRES, FRAG: 0.57.

9 A lower consistency threshold implies that conditions with lower consistency scores will be included in the solution. For example, a consistency threshold of 0.7 dismisses conditions with consistency lower than 0.7.

## Code availability

Upon request.
